# Penile tourniquet: the Wharton tourniquet

**DOI:** 10.1308/rcsann.2024.0045

**Published:** 2024-07-31

**Authors:** S Nour, GH Lafford, SM Wharton

**Affiliations:** Russells Hall Hospital, UK

## Introduction

In hypospadias repair surgery, penile tourniquets are used to create a bloodless field, induce an artificial erection, aid in the identification of fistulae and pressure test after closure. Numerous techniques have been described to create the required tourniquet effect. Those include (but are not limited to) the use of red rubber catheters, Penrose tubes, surgical gloves and rubber bands.

## Technique

**Figure 1:**
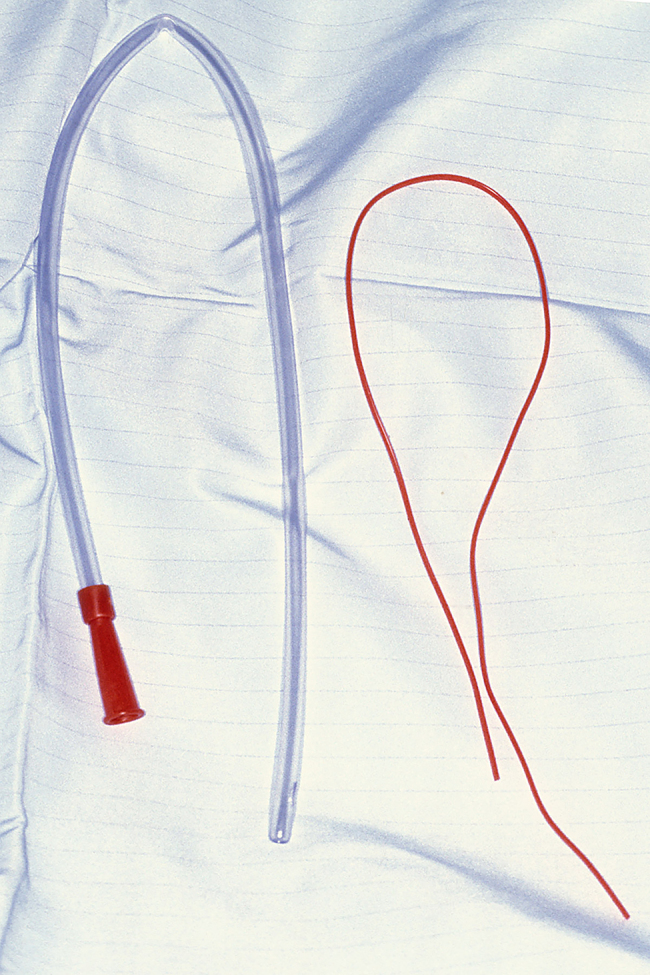
18 Fr suction catheter (left), vascular sloop (right)

The technique we describe is designed by Mr Simon Wharton. It is used regularly in our Plastic Surgery department and has been adopted in at least two other units. We use a sterile large size 18 Fr suction catheter, a vascular sloop (Figure 1) and an artery clip, all of which are available routinely in the majority of surgical units and trays.

**Figure 2:**
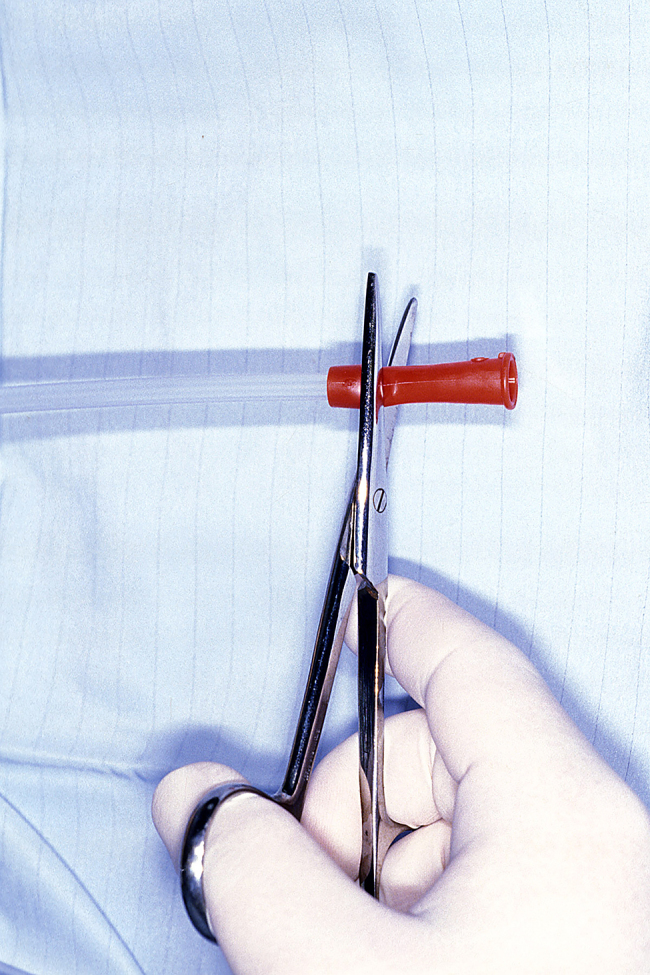
End usually attached to suction is shortened, leaving a 1cm cuff

**Figure 3:**
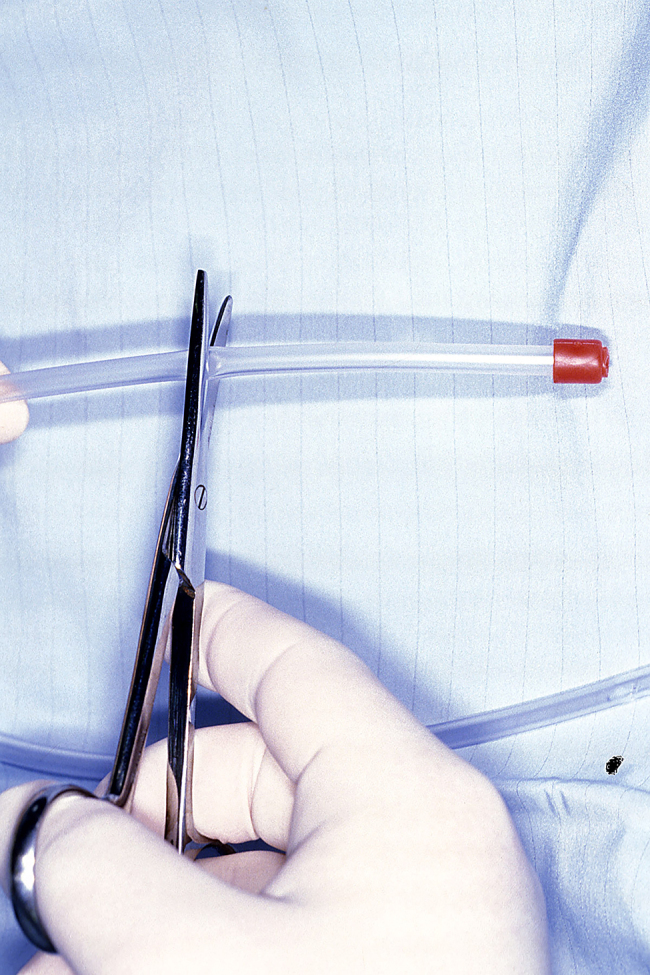
Clear end shortened to about 10cms

**Figure 4:**
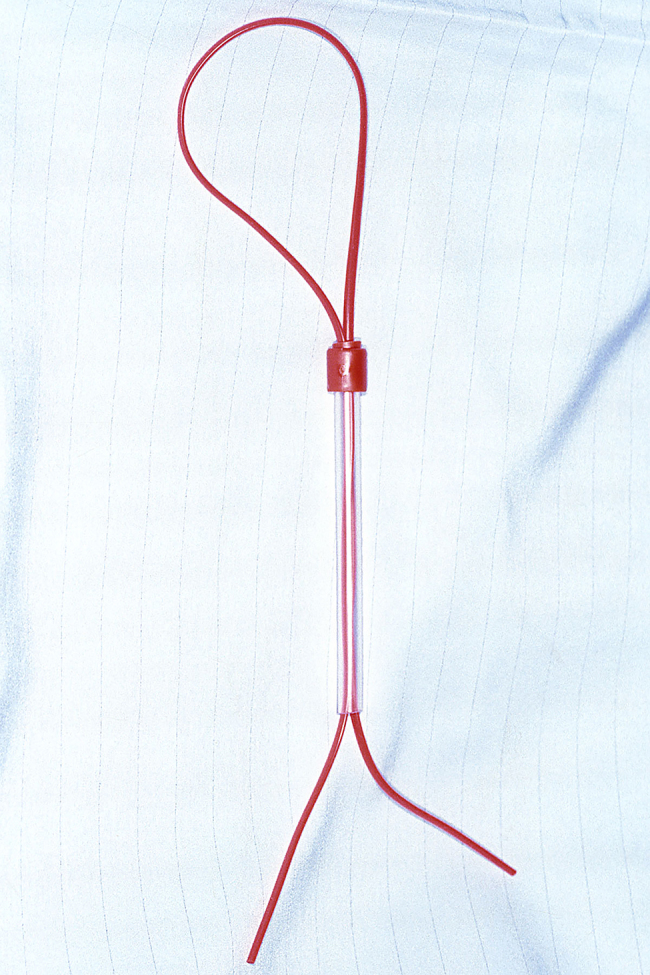
The vascular sloop is drawn through the suction tube to form a loop with two tails at the opposite end.

The suction tube is cut short at both ends using large Mayo scissors. The end normally attached to suction is shortened to leave a 1cm cuff (Figure 2). The clear end is shortened to about 10cms (Figure 3). The vascular sloop is folded in half to form a loop. A small skin hook is passed down the tube from the red end and catches on to the loop of sloop. The sloop is then drawn through the suction tube to form a loop with two tails at the opposite end (Figure 4).

**Figure 5:**
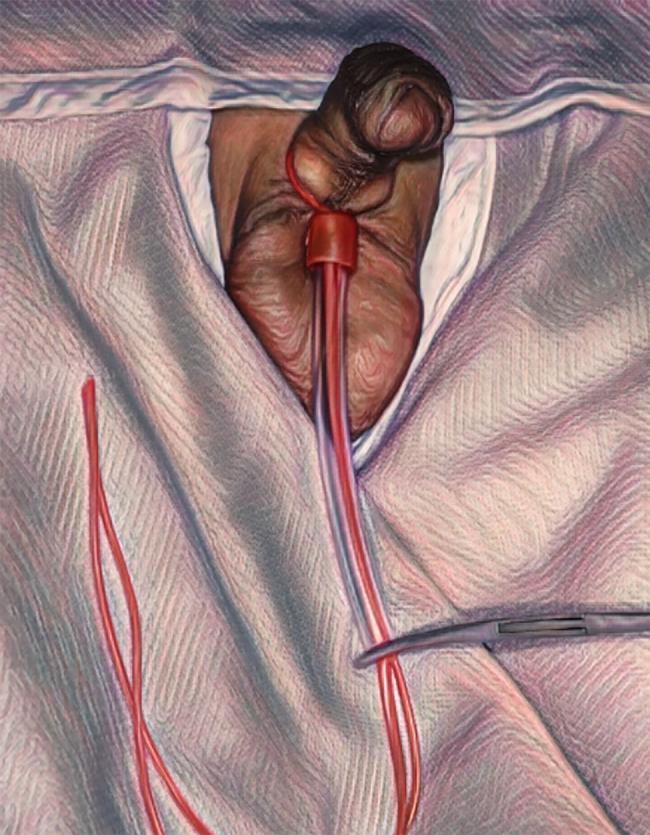
The loop is passed over the penis, stretched at both ends to create a tourniquet effect and secured with an artery clip.

The loop is passed over the penis and placed around the base. Both ends of the vascular sloop are stretched to create the required tourniquet effect around the penis and anchored to the shortened end of the suction catheter using an artery clip (Figure 5). This creates a secure clamp. The pressure can be adjusted by increasing or decreasing the tension of the vascular sloop by adjusting where the artery clip sits.

The tourniquet is released by removing the artery clip then suction tube followed by the vascular sloop.

## Discussion

Based on our experience this is a safe technique that has been used for well over ten years with no complications or failures.

